# Chimpanzee’s in Black: Visual Search for the Conspecific Body Silhouette by Chimpanzees (*Pan troglodytes*)

**DOI:** 10.1162/OPMI.a.38

**Published:** 2025-10-29

**Authors:** Masaki Tomonaga, Tomoko Imura

**Affiliations:** School of Psychological Sciences, University of Human Environments, Matsuyama, Japan; Faculty of Integrated Arts and Social Sciences, Japan Women’s University, Tokyo, Japan

**Keywords:** body perception, silhouette, chimpanzees, visual search

## Abstract

Like faces, we obtain various information from the body, such as species, individual identity, age, sex, action, and emotional state. Bodies are processed in a specialized manner, similar to faces. Furthermore, it is known that faces capture attention and cause efficient search in primates including humans (i.e., shallower response-time slopes with increasing set size). In this study we aimed to examine from a comparative cognitive perspective whether bodies have a similar effect. To this end, we conducted a visual search task using body silhouette stimuli with chimpanzees and humans as participants. The results showed that chimpanzees detected body silhouettes among a set of miscellaneous object silhouettes more quickly and accurately compared to the other stimulus categories. These results could not be explained by physical features such as pattern complexity and suggest that chimpanzees categorically perceive the bodies of their own species. In contrast, the body-specific inversion effect, which could be evidence for specialized processing of bodies, was not observed. Furthermore, in a generalization test using silhouettes of quadrupedal animals, which were novel but similar in pattern to chimpanzees, and silhouettes of humans, which were familiar but not quadrupedal, their performance was better with the silhouettes of quadrupedal animals than with the familiar human silhouettes. These findings contrast with those in humans, who efficiently searched for both chimpanzee and human silhouettes, and suggests species differences in the effect of familiarity (expertise).

## INTRODUCTION

Humans extract various kinds of information from others’ bodies (de Gelder, 2009; Peelen & Downing, 2007). We not only communicate with “intentional” gestural signs but also detect subtler cues from bodily movements and postures (Coulson, 2004; Dael et al., 2012; Goldin-Meadow, 1999; Kendon, 1994, 2017; Mehrabian, 2017; Scheflen, 1964). Furthermore, beyond these dynamic cues, the body’s shape alone allows us to infer a range of attributes about an individual, including species, identity, age, and gender (e.g., Collins, 1991; Ghuman et al., 2010).

Needless to say, in addition to bodies, faces are also crucial for our social lives. As is widely known, faces are processed in a specialized manner, favoring configural processing based on the spatial layout of features rather than on individual features (see Farah et al., 1998; Maurer et al., 2002 for review). This reliance on configural information can impair recognition when such cues are disrupted, as demonstrated by the face inversion effect (Farah et al., 1995; Valentine, 1988; Yin, 1969), the Thatcher illusion (Thompson, 1980), and the composite face effect (Calder et al., 2000; Young et al., 1987). Additionally, faces are detected efficiently in visual search tasks (Hershler & Hochstein, 2005; i.e., shallower response-time slopes with increasing set size), as defined in classic models of visual search (e.g., Treisman & Gelade, 1980; Wolfe, 1994, 2007; Wolfe et al., 1989), and robustly capture attention (Langton et al., 2008).

Many studies using body stimuli in humans strongly suggest that, like faces, bodies are processed in a specialized manner (e.g., Aviezer et al., 2012; see also Endo, 2015 for review). For instance, the inversion effect observed with faces also occurs when bodies are used as stimuli (Reed et al., 2003, 2006; Troje & Westhoff, 2006). Furthermore, in visual search tasks involving videos or photos of people and machines, a search asymmetry has been reported: human targets are detected more efficiently than machine targets when human distractors are present (Mayer et al., 2015). Additionally, in natural scenes, bodies without faces are detected as efficiently as faces without bodies (Bindemann et al., 2010). Bodies, like faces, readily capture spatial attention (Downing et al., 2004; Ro et al., 2007). In humans, specific brain regions responsible for face processing (e.g., the fusiform face area, FFA; Kanwisher & Yovel, 2006; Tong et al., 2000) are paralleled by regions for body processing, such as the extrastriate body area (EBA; Downing et al., 2001; Peelen & Downing, 2007; Thorat & Peelen, 2022) and fusiform body area (FBA; Peelen & Downing, 2005; Schwarzlose et al., 2005; Thorat & Peelen, 2022), which are involved in processing both whole bodies and individual body parts. Furthermore, the recognition of actions through body cues is thought to involve the mirror neuron system (Buccino et al., 2004; Rizzolatti & Craighero, 2004).

In nonhuman primates, information conveyed by the body is also as crucial as facial cues for maintaining social interactions. Over recent decades, intentional gestural communication has been intensively studied in great apes (Cartmill & Byrne, 2010; Genty et al., 2009; Hobaiter & Byrne, 2011, 2014; Hobaiter et al., 2022; Leavens et al., 2004; Pika & Mitani, 2006). Additionally, chimpanzees can recognize the emotional states of others not only from facial expressions but also from bodily movements (Kano & Tomonaga, 2010).

As numerous studies have shown, facial processing is also specialized in nonhuman primates (see Adachi & Tomonaga, 2017 for review). In chimpanzees, in particular, the inversion effect is clearly observed (Dahl et al., 2013; Parr et al., 1998; Tomonaga, 1999, 2007; Tomonaga & Imura, 2015), and the composite face effect has also been reported (Parr et al., 2006; Taubert et al., 2012). Moreover, faces robustly capture the attention of chimpanzees, who search for faces more efficiently than for other types of stimuli (Tomonaga & Imura, 2009, 2015).

Although face perception and recognition have been extensively studied in nonhuman primates, comparative cognitive research on body perception remains relatively limited. For instance, chimpanzees can predict movement direction based on body orientation (Tomonaga & Imura, 2023) and are capable of distinguishing between biological motion, such as a walking chimpanzee, and random motion patterns (Tomonaga, 2001). They exhibit a limited, but context-dependent sensitivity to biomechanical properties of the body (Sato et al., 2021). Furthermore, in a series of studies, Gao and colleagues (Gao et al., 2020; Gao & Tomonaga, 2018, 2020a, 2020b) reported a body inversion effect in chimpanzees, demonstrating that inverted bodies are difficult for them to perceive (cf. Matsuno & Fujita, 2018). This finding suggests that chimpanzees may process body configurations in a holistic or configural manner.

In the present study, we sought to expand our understanding of body perception in chimpanzees by employing visual search tasks using body stimuli. As noted previously, both humans and chimpanzees can efficiently detect faces among various distractors. In humans, quick and accurate detection has also been reported when body stimuli are used. Therefore, in this study, we examined whether chimpanzees could similarly detect conspecific body silhouettes quickly and accurately.

In this study, we utilized silhouette stimuli to minimize the influence of facial information on body perception. If quick and accurate search were observed for full-body images, it would be challenging to determine whether this effect was driven by the body or the face (Axelsson et al., 2019; Brandman & Yovel, 2010; Yovel et al., 2010). However, in humans, attention is known to be directed toward bodies even when they lack heads (Bindemann et al., 2010), and body silhouettes are also effective in capturing attention (Downing et al., 2004).

In Experiment 1, we first compared search performance for chimpanzee body silhouettes with that for other target categories (fish, chairs, ships) to determine whether body stimuli would lead to faster and more accurate detection. Experiment 2 examined the effect of inversion on visual search for body stimuli. If bodies are processed configurally, as faces are, then inverted body silhouettes may result in slower or less accurate detection, as shown in studies on face perception (Tomonaga & Imura, 2015). In Experiment 3, we conducted a generalization test using silhouettes of quadrupedal animals and humans to further assess the range and limitations of body-related search performance. Finally, in Experiment 4, we conducted a positive-control experiment with human participants to validate the experimental procedures used in this study.

## GENERAL METHODS

### Participants

Seven chimpanzees (*Pan troglodytes*) participated in the present study; Ayumu (male, 7 years old at the onset of the study; Great Ape Information Network (GAIN; https://shigen.nig.ac.jp/gain/), ID#0608), Cleo (female, 7 years old, #0609; see Figure 3), Pal (female, 7 years old, #0611), Pan (female, 23 years old, #0440), Chloe (female, 26 years old, #0441), Pendesa (female, 30 years old, #0095), and Ai (female, 31 years old, #0434). They had been participating in various computer-controlled perceptual and cognitive experiments including the visual search tasks (Matsuzawa, 2006; Tomonaga, 2001, 2010a, 2010b; Tomonaga et al., 2003). All chimpanzees lived in a group of 14 individuals in indoor and outdoor compounds (770 m^2^) at the Primate Research Institute, Kyoto University (Matsuzawa, 2006). During the present study, we did not conduct food and water deprivations.

In addition to chimpanzees, seven humans participated in Experiment 4. Details are described later.

### Ethics Statements

The care and use of the chimpanzees adhered to the 2nd edition of the *Guide for the Care and Use of Laboratory Primates* of the institute. The experimental designs of the present study with chimpanzees (Experiments 1–3) were approved by the institute’s Animal Welfare and Animal Care Committee (07-1544, 08-1659). All procedures also adhered to the *Guideline of the Animal Experimentation* of the Japanese Society of Animal Psychology, *Guideline for the Care and Experimental Use of Captive Primates* of the Primate Society of Japan, *Code of Ethics and Conduct* of the Japanese Psychological Association, and Japanese *Act on Welfare and Management of Animals*.

Experiments with humans (Experiment 4) adhered to the *Code of Ethics and Conduct* of the Japanese Psychological Association. Informed consent was obtained from each human participant.

### Experimental Setting

All experimental sessions were conducted in a booth (1.8 × 2.15 × 1.75 m) in a laboratory adjacent to the chimpanzee facility. The chimpanzees came to the booth through the overhead pathway connecting the facility to the booth. Two sets of 17-inch LCD monitors (I-O Data LCD-AD172F2-T, 1280 × 1024 pixels, pixel size: 0.264 mm × 0.264 mm) with touch panels were installed on the booth wall. The viewing distance was approximately 40 cm. A universal feeder (Biomedica BUF-310) outside the booth delivered small pieces of apple as food rewards through a tube connected to a food tray placed inside the booth. The computer controlled all equipment and experimental events.

### Stimuli

**Figure 1.**
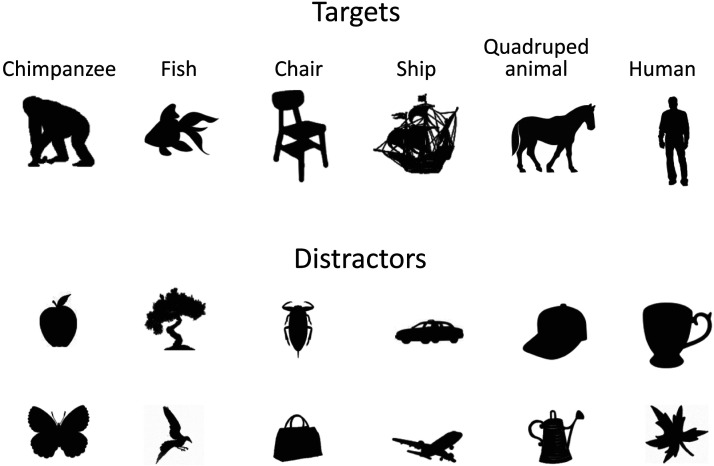
Examples of stimuli used in the present study. The top row shows target stimuli (one example from each category), and the bottom rows show distractor stimuli.

In the present study, black silhouette stimuli on a white background were used (Figure 1). The categories for the target stimuli in the visual search task included chimpanzees, fish, chairs, ships (sailing ships), humans, and quadrupedal animals. Additionally, 19 categories were prepared for the distractor stimuli. These categories consisted of both man-made objects (e.g., cars, bags, cups) and natural objects (e.g., fruits, trees, insects). For each category, multiple distinct stimuli were prepared (range: 20–138). Each stimulus was 150 × 150 pixels in size, containing an average of 3227 black pixels (*SD* = 1646). Some stimuli were made from photographs using Photoshop®, while others were selected from copyright-free archives.

### Visual Search Task

**Figure 2.**
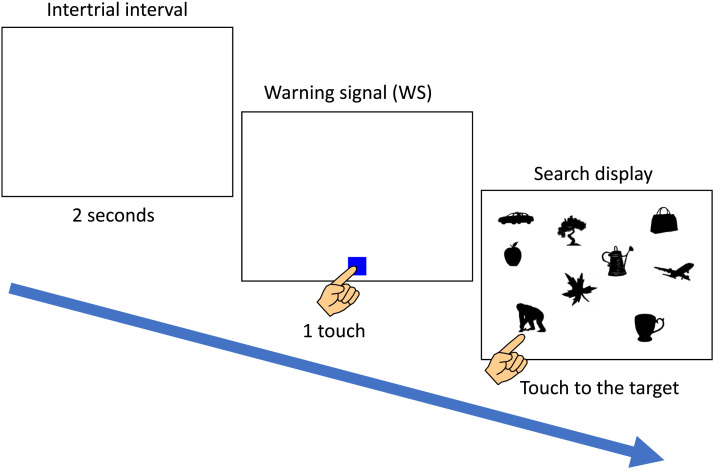
Flow of a visual search trial.

In this study, a visual search task was employed. Figure 2 shows a typical trial flow. After a 2-second intertrial interval, a warning signal (WS, a blue square, 90 × 90 pixels) was displayed randomly at the bottom of the monitor on a white background, accompanied by a sound. Once the chimpanzee touched the WS, it disappeared, and the search display appeared. The search display consisted of one target and multiple distractors, which were randomly positioned across 5 × 4 possible locations. The number of items in the display, referred to as the set size, varied randomly between trials. When the chimpanzee selected one of the stimuli, all items on the screen disappeared. A correct response (touching the target) triggered a chime and a food reward (a piece of apple), while selecting a distractor resulted in a buzzer sound. If no response was made within 8 seconds, a click sound was played, the screen was turned off, and the experiment proceeded to the next trial. These instances were counted as incorrect trials. In this experiment, we adopted a correction procedure: after an error, the same trial was presented again. If an incorrect response occurred again, only the target stimulus was presented in the next correction trial. Generally, one session was conducted per day, up to five days a week.

### Data Analysis

In the present study, we used error rates and response times for data analyses. Response times were defined as the duration between the onset of the search display and the participant’s response. Only response-time data from correct trials were used, and response times shorter than 200 ms were excluded from the analyses.

We applied generalized linear mixed model (GLMM) analyses to account for the hierarchical structure of the data. Specifically, multiple testing sessions were conducted for each of several chimpanzees, resulting in two sources of random effects: participants (chimpanzees) and sessions nested within participants. Given this data structure, GLMMs provide a more appropriate analytical framework than traditional analyses of variance. A binomial distribution was used for error rate data, and a normal distribution was applied to log-transformed response time data.

We adopted a model selection approach, in which we computed Akaike’s Information Criterion (AIC) for all possible models, ranging from the full model (including all fixed effects and their interactions) to the null model (including none), as well as all intermediate models. The model with the lowest AIC was selected as the best-fitting model (Burnham & Anderson, 2002). If the difference in AIC (ΔAIC) between models was greater than 2, the model with the smaller AIC was considered the best model. For all the main results in the present study, the ΔAICs of the selected models were greater than 2.

Although the model selection approach does not fundamentally rely on significance testing, we additionally reported test statistics, uncorrected *p*-values, and Holm-corrected *p*-values, along with 95% confidence intervals (CIs), for reference. For parameter estimates from target-category comparisons, we applied multiple comparison corrections using the Holm method and reported the corrected CIs in the result tables.

All analyses were conducted using the *lmerTest* (Kuznetsova et al., 2017) and *MuMIn* (Bartoń, 2022) packages in R version 4.2.0 (R Core Team, 2022).

## EXPERIMENT 1: VISUAL SEARCH FOR SILHOUETTE STIMULI

In Experiment 1, the chimpanzees were trained in a visual search task using silhouette stimuli, and differences in performance across target stimulus categories were examined.

### Methods

#### Stimuli.

In Experiment 1, the target stimulus categories included the silhouettes of whole-body chimpanzees, fish, chairs, and ships. Distractor stimuli were selected from all distractor categories.

#### Procedure.

##### Preliminary Training.

**Figure 3.**
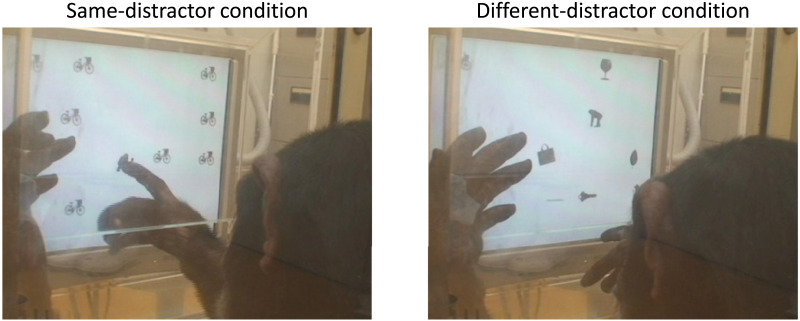
Chimpanzee Cleo performing the visual search task. Left: Same-distractor condition. Right: Different-distractor condition. In both images, she searched for a chimpanzee silhouette.

The experiment was conducted in four phases. Since all chimpanzees had extensive experience with visual search tasks prior to this study, they were already familiar with the flow of the visual search task. Therefore, in the first phase, we initially introduced a preliminary training under the condition where the distractor stimuli were identical to each other (same-distractor condition, left of Figure 3) and the number of stimuli presented on the screen (set size) was fixed at 4. Each session consisted of 40 trials, where only one target stimulus category was presented, but a different stimulus from that category was shown on each trial. The distractor category was randomly selected for each trial. The order of target stimulus categories was randomized across sessions. Due to simultaneous participation in multiple experiments, the number of sessions varied depending on the schedule of each chimpanzee, but on average, each received 3.0 sessions (range: 3–4) of this training for each target stimulus category.

In the second phase, the set size randomly varied between 5, 10, and 20 within a session. Each session consisted of 60 trials, and similar to Phase 1, only one target stimulus category was presented throughout the session. The distractor stimuli were all identical. On average, each chimpanzee underwent 5.1 sessions (range: 5–6) in this phase.

From the third phase onward, the different-distractor condition, in which the distractor stimuli were all from different categories, was introduced (right of Figure 3; see also Figure 2). In this phase, each session consisted of 40 trials, and the set size was fixed at 10. Half of the trials in each session were the same-distractor trials, while the remaining trials were the different-distractor trials. These two types of trials were presented alternately. The same-distractor trials were included to implicitly cue the target stimulus category in each session. On average, 9.1 sessions (range: 8–10) were conducted for each chimpanzee in this phase.

##### Main Experiment.

After completing all phases of the preliminary training, the chimpanzees proceeded to the main experiment, where the Different Distractor condition was applied. In each session, the set size randomly varied between 5, 10, and 20. Each session consisted of 60 trials, with the same-distractor and different-distractor trials presented alternately in equal numbers. On average, 10.1 sessions (range: 9–11) were conducted for each chimpanzee.

### Results

#### Preliminary Training.

In the first phase of the preliminary training, the mean error rates for each target stimulus category were 7.4% (SEM = 1.3) for chimpanzee, 14.5% (2.3) for fish, 10.2% (2.7) for chair, and 5.1% (0.9) for ship. The mean response times for correct trials were 1052 ms (SEM = 88) for chimpanzee, 1232 ms (79) for fish, 1213 ms (116) for chair, and 1012 ms (68) for ship.

In the second phase, where the set sizes were varied, the mean error rates and correct response times averaged across different set sizes were 0.7% (SEM = 0.2) and 972 ms (62) for chimpanzee, 4.4% (0.7) and 1187 ms (80) for fish, 2.5% (0.6) and 1098 ms (91) for chair, and 1.7% (0.4) and 955 ms (68) for ship.

In the third phase, where different-distractor trials were introduced, the mean error rates and correct response times for the different-distractor trials were 9.2% (2.2) and 1342 ms (75) for chimpanzee, 54.2% (2.3) and 1962 ms (71) for fish, 53.5% (6.2) and 2007 ms (105) for chair, and 25.9% (3.8) and 1449 ms (83) for ship.

Across the preliminary training phases, the chimpanzees generally showed lower error rates and faster response times when the target was either chimpanzee or ship, compared to fish or chair (see also Table S1 for summary of the results). No further analyses of the preliminary training results were conducted here, but the results of statistical analyses are provided in Table S2.

#### Main Experiment.

**Figure 4.**
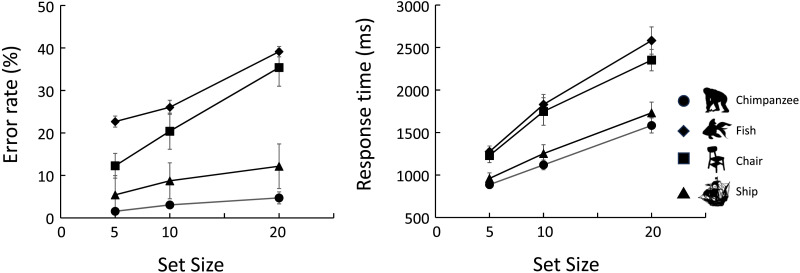
Results of Experiment 1. Left: Error rates. Right: Response times in correct trials. Error bars indicate standard errors. Only the results from the different-distractor condition are shown. The results from the same-distractor condition are presented in Table S1.

Figure 4 shows the results of the different-distractor trials. The left panel presents the mean error rates, while the right panel shows the mean correct response times. Note that the results and (Table S1) statistical analyses (Table S3) of the same-distractor trials in the main experiment are presented in the supplementary tables. The chimpanzees showed the fastest and most accurate search performance when the target was a chimpanzee body silhouette, followed by the ship condition. In contrast, when the target stimulus was either a fish or a chair, error rates increased and response times were longer compared to the other two conditions.

Generalized linear mixed models (GLMMs) were applied to analyze these data. The fixed effects were the type of target category and the set size. The results of these analyses are shown in Table 1. The full model, which included both fixed effects and their interaction, was selected for the error rates, while the selected model for the response times included only the two fixed effects without their interaction. Although there appeared to be small differences between chimpanzee and ship, and between fish and chair, the parameter estimates for all comparisons among the target categories differed significantly from zero for both error rates and response times.

**Table 1.** Summary of GLMMs for different-distractor trials in Experiment 1

Error rates											
Fixed effects	Levels	Estimate	*SE*	*Z*	*p*		corrected *p*	95% CI		95% CI (corrected)	
Set size at	Chimp	0.067	0.019	3.470	5.2E−04		5.2E−04	0.029	0.105	0.029	0.105
	Fish	0.059	0.008	7.416	1.2E−13		3.6E−13	0.043	0.075	0.040	0.078
	Chair	0.093	0.009	10.602	0		0	0.076	0.111	0.071	0.115
	Ship	0.056	0.013	4.510	6.5E−06		1.3E−05	0.032	0.081	0.028	0.084
Target	Chimp vs. Fish	2.804	0.332	8.441	0		0	2.153	3.455	1.928	3.681
	Chimp vs. Chair	1.987	0.339	5.859	4.7E−09		1.9E−08	1.322	2.652	1.140	2.834
	Chimp vs. Ship	1.298	0.363	3.575	3.5E−04		7.0E−04	0.586	2.009	0.484	2.111
	Fish vs. Chair	−0.817	0.191	4.272	1.9E−05		5.8E−05	−1.192	−0.442	−1.275	−0.359
	Fish vs. Ship	−1.506	0.231	6.523	6.9E−11		3.4E−10	−1.959	−1.054	−2.101	−0.911
	Chair vs. Ship	−0.689	0.241	2.862	4.2E−03		4.2E−03	−1.161	−0.217	−1.161	−0.217

Response times											
Fixed effects	Levels	Estimate	*SE*	*df*	*t*	*p*	corrected *p*	95% CI		95% CI (corrected)	
Set size		0.037	0.001	7100.0	40.217	0	–	0.035	0.038	–	–
Target	Chimp vs. Fish	0.405	0.019	267.3	20.785	9.8E−58	5.9E−57	0.367	0.443	0.350	0.461
	Chimp vs. Chair	0.331	0.019	250.7	17.298	1.3E−44	5.1E−44	0.293	0.367	0.278	0.383
	Chimp vs. Ship	0.053	0.019	223.1	2.839	4.9E−03	4.9E−03	0.016	0.088	0.011	0.095
	Fish vs. Chair	−0.075	0.020	304.2	3.682	2.7E−04	5.5E−04	−0.114	−0.035	−0.126	−0.024
	Fish vs. Ship	−0.353	0.020	275.5	17.831	8.6E−48	4.3E−47	−0.391	−0.314	−0.409	−0.297
	Chair vs. Ship	−0.278	0.019	258.5	14.331	1.2E−34	3.5E−34	−0.316	−0.240	−0.330	−0.226

*Note*. Chimp: Chimpanzees, SE: standard error, CI: confidence interval. Corrected *p*-values and CIs based on Holm’s method are also shown.

### Discussion

The results of Experiment 1 revealed that the chimpanzees searched for the full-body silhouette of a chimpanzee more quickly and accurately than targets from the other categories. This finding was consistent with our prediction. However, a more detailed analysis of the results indicated that not only chimpanzees but also ships were searched more quickly and accurately than the other two categories. Ships are an unfamiliar stimulus category for the chimpanzees, as they have never encountered them in their daily lives. One possible reason for their quick setection of such unfamiliar stimuli may lie not in the semantic attributes of the category, but rather in more basic visual features.

**Figure 5.**
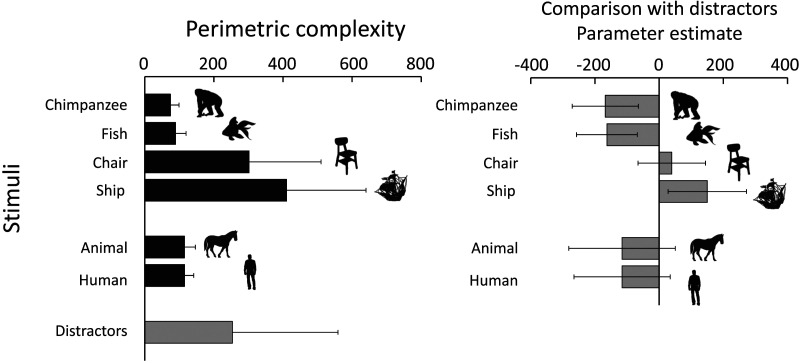
Left: Mean perimetric complexity for each stimulus category. Error bars indicate standard deviations. Right: Estimated differences in perimetric complexity between the target category and the distractor stimuli. Error bars represent corrected 95% CIs.

At first glance, ship stimuli appear more complex than those in categories like chimpanzee or fish. Therefore, an exploratory analysis using complexity as an index was conducted. We adopted perimetric complexity (Arnoult & Attneave, 1956; Saito et al., 2022) as the index in the present study, defined as the square of the perimeter of a silhouette divided by the area of the black region (i.e., the total number of pixels). Left of Figure 5 shows the mean and standard deviation of perimetric complexity for each category. For distractors, all sub-categories were pooled together to calculate the mean and standard deviation. As shown in this graph, stimuli in the ship category had higher perimetric complexity compared to the other categories.

We conducted a generalized linear model (GLM) analysis on these data. A portion of the results is shown on the right of Figure 5 (the full results are presented in Table S4). Each bar represents the estimated difference in perimetric complexity between the target category and the distractor stimuli. Negative values indicate that the distractors were more complex than the target categories, whereas positive values indicate that the targets were more complex. Error bars represent corrected 95% CIs.

This analysis revealed that the chimpanzee and fish categories were less complex than the distractors. In contrast, the ship category was more complex than the distractors. Additionally, for the chair category and for the quadrupeds and humans used in Experiment 3, there was no significant difference in complexity between targets and distractors.

Previous studies have shown that visual search is less efficient when the similarity between stimuli is high (e.g., Duncan & Humphreys, 1989). Other studies have also suggested that the stimulus complexity influences visual search performance (Marsh et al., 2021; Sun & Firestone, 2021). In particular, search asymmetry has been reported along the dimension of complexity, such that it is generally easier to detect a complex shape among simpler distractors than vice versa (Sun & Firestone, 2021).

Based on the analysis of the stimulus complexity, the findings for the chair category may reflect the effect of similarity between the target and distractors. In contrast, the findings for the ship and fish categories may be interpreted as instances of search asymmetry along the complexity dimension. However, the results for the chimpanzee category cannot be readily explained by differences in complexity. If complexity were the only factor, the relatively simple silhouette of a chimpanzee should have been more difficult to detect. Nevertheless, the fact that the chimpanzees performed more quickly and accurately for the full-body silhouette of the chimpanzee strongly suggests that they may have processed it differently from the other stimuli, potentially recognizing it as a “chimpanzee.”

**Figure 6.**
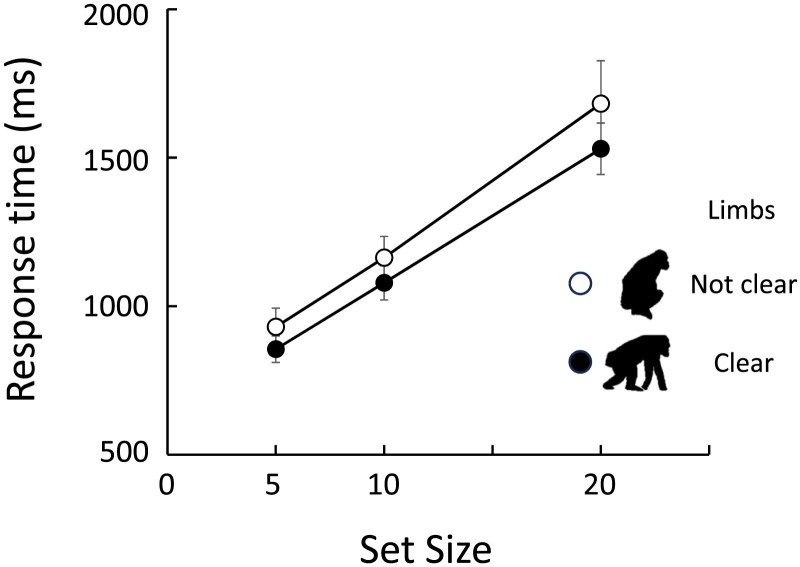
Effect of limb clarity on response times in chimpanzee-target trials in Experiment 1.

If the chimpanzees perceived the silhouettes of chimpanzees as belonging to a distinct category, what specific cues facilitated this discrimination? We focused on the limbs and classified the 38 chimpanzee silhouettes presented in this experiment into those with clearly identifiable limbs (*N* = 22) and those without (*N* = 16). GLMMs were applied to analyze error rates and correct response times for chimpanzee target stimuli, with limb clarity and set size as fixed effects. No significant difference in error rates was found between clarity types (Clear = 2.3%, Not Clear = 2.8%), and the model including only set size was selected (*β* = 0.067, [0.026, 0.107]). In contrast, correct response times were significantly faster for stimuli with clearly identifiable limbs compared to those without (Figure 6; limb clarity, *β* = 0.060, [0.024, 0.097]; set size, *β* = 0.034, [0.031, 0.037]). Gao et al. (2020) reported in their study on body perception in chimpanzees, using a matching-to-sample task, that scrambling the first-order configuration of the arms, legs, and head eliminated the body inversion effect. Taken together, these findings suggest that the natural spatial arrangement of limbs may be a key cue for detecting the silhouette of a chimpanzee. This issue is further examined in Experiment 3.

Chimpanzees searched for the silhouette of a chimpanzee’s body better than for other stimulus categories, suggesting that they processed the chimpanzee silhouette in a manner distinct from other stimuli. This raises the question: Did they process the silhouette holistically, as they do with facial stimuli (Aviezer et al., 2012; Farah et al., 1998; Maurer et al., 2002)? One critical test for examining holistic processing is to assess changes in performance when stimuli are presented in an inverted orientation (Gao & Tomonaga, 2018; Matsuno & Fujita, 2018; Reed et al., 2003, 2006). Therefore, Experiment 2 was designed to investigate this “inversion effect.”

## EXPERIMENT 2: INVERSION EFFECT

### Methods

The same chimpanzees who participated in Experiment 1 also took part in Experiment 2. In this experiment, the stimulus set from Experiment 1 (upright stimuli) was expanded to include inverted versions of these stimuli. The set size was fixed at 10. As in Experiment 1, same-distractor and different-distractor trials were alternately presented. Within each session, only a single target stimulus category appeared, but the orientation (upright or inverted) varied randomly by trial. In inverted trials, both target and distractor stimuli were presented in the inverted orientation. Each session consisted of 40 trials, with upright and inverted trials presented equally. Each chimpanzee completed an average of 10.3 sessions (range: 10–11) for each target stimulus category.

### Results and Discussion

**Figure 7.**
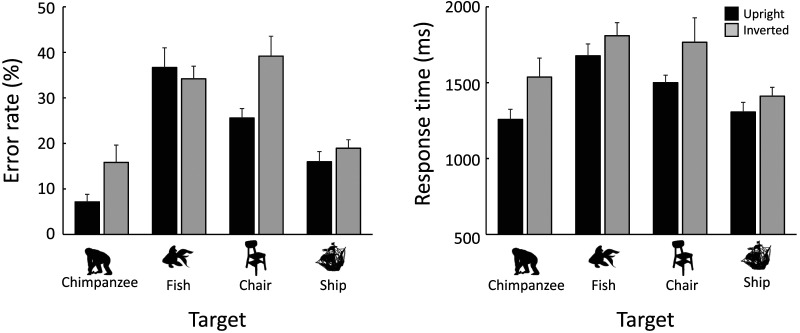
Results of Experiment 2. Left: Error rates. Right: Response times in correct trials. Black bars represent the upright condition; gray bars represent the inverted condition. Error bars indicate standard errors.

As in Experiment 1, the results for the same-distractor trials are shown in Tables S1 and S3. Figure 7 shows the mean error rates (left panel) and the mean correct response times (right panel) for each target stimulus category and orientation condition in the different-distractor trials. With the exception of the error rates for the fish and ship targets, the upright targets were detected quickly compared to inverted stimuli, regardless of the target category. The GLMM results supported these visual inspections, with the full model, including interaction, selected for error rates and the model without interaction selected for correct response times (Table 2).

**Table 2.** Summary of GLMMs for different-distractor trials in Experiment 2

Error rates											
Fixed effects	Levels	Estimate	*SE*	*Z*	*p*	corrected *p*		95% CI		95% CI (corrected)	
Orientation at	Chimp	0.879	0.190	4.625	3.7E∓06	1.1E∓05		0.506	1.251	0.424	1.334
	Fish	−0.128	0.115	1.117	2.6E−01	5.3E−01		−0.353	0.097	−0.386	0.129
	Chair	0.661	0.116	5.688	1.3E−08	5.1E−08		0.434	0.889	0.371	0.952
	Ship	0.150	0.144	1.040	3.0E−01	3.0E−01		−0.133	0.432	−0.132	0.432
Target at Up	Chimp vs. Fish	2.177	0.180	12.101	0	0		1.824	2.529	1.702	2.651
	Chimp vs. Chair	1.604	0.183	8.770	0	0		1.245	1.962	1.133	2.075
	Chimp vs. Ship	1.002	0.191	5.237	1.6E−07	4.9E−07		0.627	1.376	0.544	1.459
	Fish vs. Chair	−0.573	0.126	4.537	5.7E−06	1.1E−05		−0.820	−0.325	−0.856	−0.290
	Fish vs. Ship	−1.175	0.139	8.486	0	0		−1.447	−0.904	−1.521	−0.829
	Chair vs. Ship	−0.603	0.143	4.229	2.3E−05	2.3E−05		−0.882	−0.323	−0.882	−0.323
Target at Inv	Chimp vs. Fish	1.170	0.141	8.284	2.2E−16	1.1E−15		0.893	1.447	0.806	1.534
	Chimp vs. Chair	1.387	0.139	9.977	0	0		1.114	1.659	1.020	1.754
	Chimp vs. Ship	0.273	0.152	1.790	7.3E−02	1.5E−01		−0.026	0.571	−0.069	0.614
	Fish vs. Chair	0.217	0.121	1.789	7.4E−02	7.4E−02		−0.021	0.455	−0.021	0.455
	Fish vs. Ship	−0.897	0.137	6.551	5.7E−11	1.7E−10		−1.166	−0.629	−1.225	−0.569
	Chair vs. Ship	−1.114	0.135	8.270	2.2E−16	8.9E−16		−1.378	−0.850	−1.451	−0.778

Response times											
Fixed effects		Estimate	*SE*	*df*	*t*	*p*	corrected *p*	95% CI		95% CI (corrected)	
Orientation		0.107	0.015	2586.4	7.237	6.0E−13	–	0.078	0.136	–	–
Target	Chimp vs. Fish	0.242	0.021	275.8	11.396	6.7E−25	3.4E−24	0.200	0.283	0.182	0.301
	Chimp vs. Chair	0.130	0.021	256.7	6.288	1.4E−09	4.1E−09	0.090	0.171	0.075	0.186
	Chimp vs. Ship	−0.021	0.020	221.7	1.055	2.9E−01	2.9E−01	−0.059	0.018	−0.065	0.024
	Fish vs. Chair	−0.111	0.023	343.6	4.907	1.4E−06	2.9E−06	−0.156	−0.067	−0.169	−0.054
	Fish vs. Ship	−0.263	0.022	288.7	12.070	2.0E−27	1.2E−26	−0.305	−0.220	−0.325	−0.200
	Chair vs. Ship	−0.151	0.021	268.7	7.109	1.1E−11	4.2E−11	−0.193	−0.110	−0.210	−0.093

*Note*. Up = upright; Inv = inverted.

If the chimpanzees processed the chimpanzee silhouette configurally but not the other target stimulus categories, the inversion effect should have occurred only for the chimpanzee silhouettes. However, this was not the case. Since primary visual features, such as perimetric complexity, remain unchanged upon inversion, these features cannot explain the present results. If familiarity with each target category in the chimpanzees’ daily lives influenced the results, the inversion effect observed for the chair target might be explained (Diamond & Carey, 1986; Yin, 1969). Indeed, the inversion effect was not observed in error rates for fish and ship targets, both of which are less familiar to the chimpanzees than chairs. However, since the inversion effect was observed for response times across all target stimulus categories, we should be cautious in interpreting these results. It is possible that familiarity with the entire set of upright silhouette stimuli increased during the experiments, leading to less quick detections for the less familiar inverted stimuli. Further investigation is needed to understand why search performance deteriorated across all target categories with inverted stimuli.

**Figure 8.**
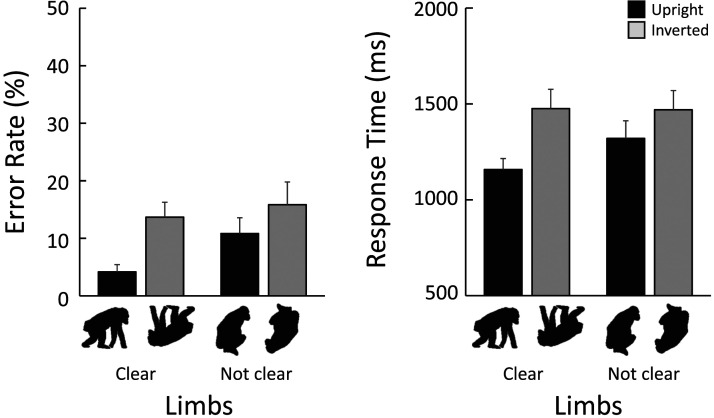
Effect of limb clarity on response times in chimpanzee-target trials in Experiment 2.

In Experiment 1, the search performance for chimpanzee silhouettes was affected by the clarity of the limbs. To explore whether the first-order spatial configuration between the limbs and body contributes to the inversion effect, we further analyzed the data from the chimpanzee silhouettes with limb clarity as an additional fixed factor in GLMMs. The results, shown in Figure 8, indicate that the effect sizes of the inversion were influenced by limb clarity for both error rates and correct response times. GLMM analyses showed that the full models, which included limb clarity, stimulus orientation, and their interaction, were selected for both error rates (limb clarity: *β* = 1.040, [0.428, 1.652], orientation: *β* = 1.312, [0.778, 1.845], interaction: *β* = −0.870, [−1.610, −0.130]) and correct response times (limb clarity: *β* = 0.142, [0.072, 0.212], orientation: *β* = 0.240, [0.182, 0.298], interaction: *β* = −0.142, [−0.240, −0.046]). Specifically, the inversion effect was more substantial when the limbs were clearly identified. This finding suggests that configural processing may play a critical role in detecting the full-body silhouette of the chimpanzee.

The results of Experiments 1 and 2 suggest that the chimpanzees may attend to limbs and their spatial arrangement when detecting chimpanzee silhouettes. At the same time, the findings from Experiment 2 suggest that familiarity might also influence the processing of silhouette stimuli. Therefore, in Experiment 3, we conducted a generalization test with novel stimulus categories to further examine the effects of perceptual cues and familiarity on processing silhouette stimuli.

## EXPERIMENT 3: GENERALIZATION TEST

### Methods

In Experiment 3, four target categories were employed: chimpanzees (20 stimuli) and chairs (20), which had been used in previous experiments, as well as quadrupedal mammals (24) and humans (31) as new target categories. For the two existing categories, new stimuli that had not been used in prior experiments were prepared. Each session consisted of 40 trials, with same-distractor and different-distractor trials presented alternately, as in previous experiments. The set size was fixed at 10, and the stimulus positions were randomized in each trial. Within each session, only one target category appeared, and the order of target categories was randomized across sessions. An average of five sessions (range: 4–6) were conducted for each target category.

### Results and Discussion

**Figure 9.**
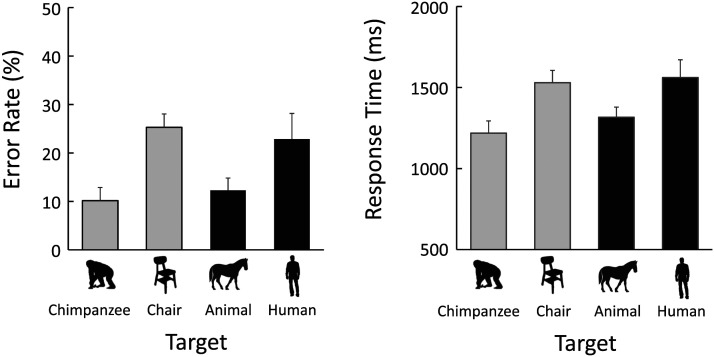
Results of Experiment 3. Left: Error rates. Right: Response times in correct trials. Error bars indicate standard errors. Gray bars represent familiar categories, and black bars represent novel (test) categories.

The results of the same-distractor trials are presented in Tables S1 and S3. Figure 9 shows the results of the different-distractor trials, with the left panel indicating the mean error rate for each target category and the right panel showing the mean correct response times. For the two previously used categories, the results were consistent with prior results: the chimpanzee target was searched more quickly and accurately than the chair. For the two new categories, the chimpanzees detected quadrupedal animals better than human silhouettes. These observations were statistically confirmed by GLMM analyses (Table 3).

**Table 3.** Summary of GLMMs for different-distractor trials in Experiment 3

Error rates											
Fixed effects	Levels	Estimate	*SE*	*Z*	*p*	corrected *p*		95% CI		95% CI (corrected)	
Target	Chimp vs. Chair	1.130	0.201	5.623	1.9E−08	1.1E−07		0.736	1.524	0.600	1.660
	Chimp vs. Animal	0.276	0.217	1.274	2.0E−01	4.1E−01		−0.149	0.701	−0.210	0.762
	Chimp vs. Human	0.970	0.209	4.642	3.5E−06	1.7E−05		0.561	1.380	0.432	1.508
	Chair vs. Animal	−0.854	0.195	4.381	1.2E−05	4.7E−05		−1.235	−0.472	−1.340	−0.367
	Chair vs. Human	−0.160	0.184	0.866	3.9E−01	3.9E−01		−0.521	0.202	−0.521	0.202
	Animal vs. Human	0.694	0.199	3.491	4.8E−04	1.4E−03		0.304	1.083	0.218	1.170

Response times											
Fixed effects	Levels	Estimate	*SE*	*df*	*t*	*p*	corrected *p*	95% CI		95% CI (corrected)	
Target	Chimp vs. Chair	0.176	0.032	154.5	5.524	1.4E−07	6.9E−07	0.114	0.239	0.048	0.305
	Chimp vs. Animal	0.079	0.031	126.9	2.522	1.3E−02	2.6E−02	0.018	0.140	0.000	0.158
	Chimp vs. Human	0.227	0.032	123.3	7.013	1.4E−10	8.2E−10	0.164	0.291	0.133	0.322
	Chair vs. Animal	−0.098	0.032	160.0	3.052	2.7E−03	8.0E−03	−0.160	−0.035	−0.183	−0.012
	Chair vs. Human	0.051	0.033	151.2	1.530	1.3E−01	1.3E−01	−0.014	0.116	−0.024	0.126
	Animal vs. Human	0.148	0.032	131.5	4.582	1.1E−05	4.2E−05	0.085	0.212	0.059	0.238

The chimpanzees more quickly and accurately searched for the silhouettes of quadrupedal animals, which are perceptually similar to the conspecific’s silhouette, than for human silhouettes, which were familiar to them. These findings are consistent with the results of the chair target in previous experiments. In the experimental environment, several types of chairs are commonly present, resulting in high familiarity to the chair category. Nevertheless, as with the familiar human target in this experiment, search efficiency for chairs was low. These results also align with the findings of Gao and Tomonaga (2018, 2020a) that in silhouette matching tasks, chimpanzees showed a body inversion effect for silhouettes resembling their own body but not for upright human silhouettes. Furthermore, in matching tasks using photo stimuli (Gao & Tomonaga, 2020b), an inversion effect was observed not only for quadrupedal animals, such as horses and macaque monkeys, but also for photographs of humans in quadrupedal postures (crawling). Taken together, these results and those of the present experiment suggest that chimpanzees are better at searching for patterns that are perceptually similar to their own silhouettes, and may have relatively greater difficulty in searching for highly familiar (upright) human silhouettes.

## EXPERIMENT 4: HUMAN EXPERIMENT

In Experiment 4, a visual search task similar to those in the previous experiments was conducted with human participants. This experiment had two main purposes. The first was to serve as a positive control to confirm that the visual search task applied to the chimpanzees in this study was appropriate for investigating body perception. The second was to examine whether human participants could search quickly for the silhouettes of chimpanzees, a species with high familiarity despite being perceptually different from their own.

### Methods

In this experiment, seven undergraduate students participated, including one male. They were enrolled in a foundational course in comparative cognition focusing on chimpanzees, during which they had the opportunity to observe chimpanzees closely. All participants had normal or corrected-to-normal vision. The sessions were conducted in the same booth and apparatus as used for the chimpanzees. Participants wore lab coats and entered a thoroughly sanitized booth for the experiment.

In addition to the four target categories used in Experiment 1 (chimpanzees, fish, chairs, and ships), human silhouettes were also presented as targets in this experiment. The visual search task was identical to the one performed by chimpanzees, except that no food rewards were given for correct responses. Each participant was verbally instructed to find the target stimulus different from the distractors on the screen. After preliminary training of 10–23 trials, the main experiment began. Each session in the main experiment consisted of 120 trials, with same- and different-distractor trials presented alternately. The set size varied randomly between 5, 10, and 20 in each trial. As in the chimpanzee experiments, the target category was fixed within each session, with a different target category presented in random order across sessions. Unlike Experiment 1, each participant completed only one session per target category.

### Results and Discussion

The results of the same-distractor trials are shown in Table S1. Accuracy in the different-distractor trials was extremely high, with a mean error rate of 1.8% (SEM = 0.4). Therefore, no further statistical analyses were conducted on the error rates.

**Figure 10.**
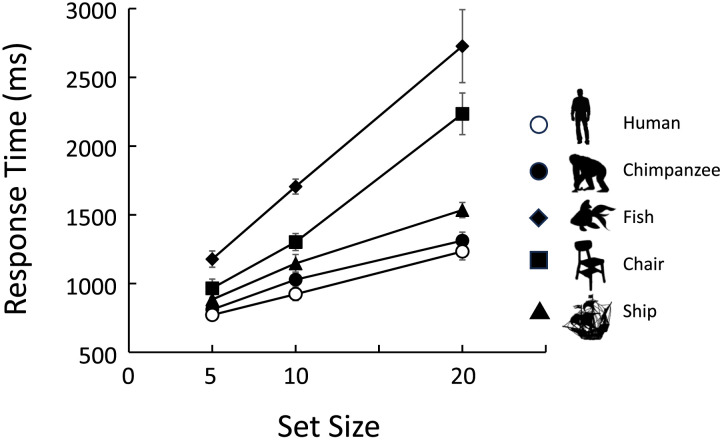
Results of the human experiment (Experiment 4). Only response times in correct trials are shown. Error bars indicate standard errors.

Figure 10 shows the results of the mean response times for correct different-distractor trials. Human participants detected the human, chimpanzee, and ship targets more quickly than the chair and fish targets. Table 4 summarizes the results of the GLMM, indicating that a model including target type and set size was selected. Parameter estimates for each target category showed that the participants were fastest to find the targets in the following order: humans, chimpanzees, ships, chairs, and fish.

**Table 4.** Summary of GLMMs for different-distractor trials in Experiment 4

Error rates											
Fixed effects	Levels	Estimate	*SE*	*df*	*t*	*p*	corrected *p*	95% CI		95% CI (corrected)	
Set size		0.036	0.002	2051	23.942	6.5E−112	–	0.033	0.039	–	–
Target	Chimp vs. Fish	0.475	0.030	2051	15.789	4.4E−53	3.9E−52	0.416	0.534	0.385	0.565
	Chimp vs. Chair	0.261	0.030	2051	8.790	3.1E−18	1.9E−17	0.203	0.320	0.176	0.347
	Chimp vs. Ship	0.106	0.030	2051	3.575	3.6E−04	7.2E−04	0.048	0.164	0.032	0.180
	Chimp vs. Human	−0.083	0.030	2051	2.798	5.2E−03	5.2E−03	−0.141	−0.025	−0.149	−0.016
	Fish vs. Chair	−0.214	0.030	2051	7.083	1.9E−12	9.7E−12	−0.273	−0.154	−0.298	−0.129
	Fish vs. Ship	−0.369	0.030	2051	12.282	1.7E−33	1.3E−32	−0.428	−0.310	−0.458	−0.280
	Fish vs. Human	−0.558	0.030	2051	18.566	3.0E−71	3.0E−70	−0.617	−0.499	−0.649	−0.467
	Chair vs. Ship	−0.156	0.030	2051	5.235	1.8E−07	5.5E−07	−0.214	−0.097	−0.234	−0.077
	Chair vs. Human	−0.344	0.030	2051	11.591	3.9E−30	2.7E−29	−0.403	−0.286	−0.431	−0.258
	Ship vs. Human	−0.189	0.030	2051	6.380	2.2E−10	8.7E−10	−0.247	−0.131	−0.270	−0.108

*Note*. Error rates were not analyzed.

The results of Experiment 4 indicated that quick search for human conspecific silhouettes was also observed in humans, demonstrating that the visual search task used in this study is suitable for examining efficient search for conspecific silhouette stimuli. Additionally, it was revealed that humans who were highly familiar with chimpanzees conducted quick searches not only for human silhouettes but also for chimpanzee silhouettes. This finding contrasts with the results of the generalization test with chimpanzees in Experiment 3 and suggests that there may be species differences in the effect of familiarity or expertise between chimpanzees and humans (Gao & Tomonaga, 2020a, 2020b).

However, for the human participants in this experiment, categories other than humans and chimpanzees were also highly familiar. Therefore, it may be difficult to attribute the present results solely to familiarity. Further investigation will be required to clarify this issue.

## GENERAL DISCUSSION

In the present study, we investigated body perception in chimpanzees using a visual search task involving a conspecific body silhouette. The results revealed that chimpanzees detected the silhouette of a conspecific body with higher accuracy and shorter response times compared to the other target stimuli. Interestingly, similar performance was also observed for the ship target, despite its unfamiliarity to the chimpanzees. It is plausible that good performances for the ship and for the chimpanzee silhouette may be driven by qualitatively different mechanisms. Additional analyses in Experiment 1 suggest that the chimpanzees’ success in detecting the ship target can be accounted for by its image complexity. Specifically, the ship silhouette used in this study exhibited higher complexity, defined by perimetric complexity, compared to the distractor stimuli, which may have served as a visual cue. This possibility is further supported by similar results obtained in the human experiment (Experiment 4).

On the other hand, in the search for the chimpanzee body silhouette, additional analysis revealed that silhouettes with clearly identifiable limbs were detected more quickly. This finding strongly suggests that the presence of limbs served as a perceptual cue for detecting conspecific bodies. Furthermore, our results are consistent with previous studies in both chimpanzees and humans, which have shown that the body inversion effect disappears when the limbs are spatially scrambled in photographs (Gao & Tomonaga, 2020a).

The present findings further suggest that quick and accurate search for bodies may involve configural processing. In this regard, similar perceptual mechanisms may underlie the efficient search for both faces and bodies (Aviezer et al., 2012; Endo, 2015). At least in humans, a parallel relationship has been observed, with distinct neural regions specialized for processing each category (e.g., Peelen & Downing, 2007).

In chimpanzees, the previous studies suggested that configural processing may be involved in the efficient search for the face (Tomonaga & Imura, 2009, 2015). If configural processing also contributes to body search, then disrupting such processing should result in worse search performance. To test this possibility, Experiment 2 examined the effect of stimulus inversion. The results showed that the search for the inverted silhouette was less accurate, regardless of the target category. The reason for this non-specific inversion effect remains unclear. Whether a similar non-specific inversion effect can be observed in humans also warrants further investigation (cf. Yin, 1969).

However, an additional analysis, similar to that conducted in Experiment 1, revealed that chimpanzee silhouettes with clearly identifiable limbs exhibited a larger inversion effect. This finding suggests that configural processing is, at least to some extent, involved in the quick and accurate search for the body.

The results of the generalization test in Experiment 3 demonstrated that quadruped animal silhouettes, which are perceptually similar to the chimpanzee body, were searched better than the silhouettes of humans, despite the latter being more familiar to the chimpanzee participants. This finding suggests that perceptual similarity has a stronger influence than familiarity on the perceptual processing of bodies in chimpanzees. Furthermore, this result is consistent with previous studies showing that perceptual similarity plays a greater role than familiarity in the body inversion effect (Gao & Tomonaga, 2020a, 2020b).

However, it should be noted that the chimpanzees in the present study were highly familiar with quadrupedal macaques in their daily environment. Thus, interpreting the current findings solely as a dichotomy between familiarity and perceptual similarity may be overly simplistic. Supporting this view, human participants in Experiment 4 also showed slower detection of the fish category, despite its familiarity. These findings suggest that familiarity alone cannot fully account for the observed differences in search performance.

One possible contributing factor is the internal structure of the target categories. The “chimpanzee” and “human” categories each consisted of exemplars from a single species, whereas categories such as “fish” and “quadrupedal animals” included multiple distinct species with considerable morphological variation. Likewise, the “chair” category comprised various subtypes with differing forms and features. These structural differences may have reduced perceptual coherence within those categories, making target detection more difficult.

According to Rosch et al. (1976), as the categorical level shifts from basic to superordinate, the perceptual similarity among members tends to decrease. It is therefore possible that the relatively poor search performance for fish and chair stimuli in both chimpanzees and humans was influenced by this difference in category level. However, the fact that chimpanzees showed differential performance between the human and chimpanzee categories cannot be fully explained by this account alone. Further investigation is required to address these unresolved issues.

The results of the present study suggest that chimpanzees search for body stimuli better than for those from other categories, and that this search performance may be driven by configural processing. However, as discussed above, several issues remain to be addressed. One important question is whether the chimpanzees really recognized the conspecific silhouettes as representing chimpanzees. One possible approach to investigate this would be to use a face–silhouette matching task (cf. Tomonaga & Kawakami, 2023). In such a task, for example, a chimpanzee face would be presented as the sample stimulus, and the participants would be required to choose the corresponding body silhouette from among several alternatives.

In a preliminary matching test conducted with the same chimpanzee participants in the present study, no strong evidence was found to suggest that they recognized the silhouettes as representing chimpanzees (Tomonaga, 2024). However, since this test had some procedural issues, further investigation is needed.

Body perception is closely related to the topics relevant to comparative cognition, such as communication, self-recognition, and species recognition, although there were not so many studies on it. Future studies involving a variety of species are needed to deepen our understanding of this important area.

## ACKNOWLEDGMENTS

We are very grateful to all the chimpanzee research and care staff for their invaluable support, technical advice, and care of the chimpanzees.

## FUNDING INFORMATION

The present study was financially supported by Grants-in-Aid for Scientific Research from the Japan Society for the Promotion of Science (JSPS) (19300091, 20002001, and 23220006).

## AUTHOR CONTRIBUTIONS

MT: Conceptualization; Data curation; Formal analysis; Funding acquisition; Investigation; Methodology; Project administration; Resources; Software; Supervision; Validation; Visualization; Writing – original draft; Writing – review & editing. TI: Conceptualization; Investigation; Resources; Validation; Writing – original draft; Writing – review & editing.

## DATA AVAILABILITY STATEMENT

The dataset used for the analyses in this study is provided as supplementary material.

## Supplementary Material


