# Supplementary material for: Chimpanzee’s in Black: Visual Search for the Conspecific Body Silhouette by Chimpanzees (Pan troglodytes)
Source: Open Mind (Camb). 2025 Oct 29;9:1802–25. doi: 10.1162/OPMI.a.38 (PMC12594578; doi:10.1162/OPMI.a.38)
Supplement: Supplementary file 1 [file opmi-09-1802-s001.pdf]

Table S1.Mean error rates and response times for correct trials in each experiment.

	Trial type	Target	Set size	Same-distractor trials				Different-distractor trials			
				Error rate	(SE)	RT	(SE)	Error rate	(SE)	RT	(SE)
Preliminary training	Upright	chimpanzee	4	7.4	(1.3)	1052	(88)				
Phase 1		fish	4	14.5	(2.3)	1232	(79)				
Set size=4		chair	4	10.2	(2.7)	1213	(116)				
SD trials		ship	4	5.1	(0.9)	1012	(68)				
Phase 2	Upright	chimpanzee	5	0.8	(0.2)	880	(51)				
Set size varied			10	0.7	(0.3)	938	(62)				
SD trials			20	0.5	(0.2)	1098	(82)				
		fish	5	4.6	(1.0)	1052	(68)				
			10	3.9	(0.9)	1173	(89)				
			20	4.7	(1.0)	1336	(87)				
		chair	5	3.2	(0.6)	978	(69)				
			10	2.0	(0.8)	1069	(86)				
			20	2.3	(0.8)	1244	(118)				
		ship	5	2.4	(0.6)	886	(53)				
			10	1.0	(0.4)	927	(82)				
			20	1.7	(0.4)	1052	(74)				
Phase 3	Upright	chimpanzee	10	0.3	(0.1)	915	(30)	9.2	(2.2)	1342	(75)
Set size=10		fish	10	3.1	(0.5)	1282	(77)	54.2	(2.3)	1964	(71)
SD & DD trials		chair	10	1.8	(0.5)	1184	(100)	53.5	(6.2)	2007	(105)
		ship	10	1.3	(0.5)	967	(83)	25.9	(3.8)	1449	(83)
Exp.1	Upright	chimpanzee	5	0.5	(0.2)	770	(35)	1.6	(0.6)	886	(46)
			10	0.3	(0.2)	886	(54)	3.1	(0.8)	1119	(58)
			20	0.1	(0.1)	971	(55)	4.7	(1.4)	1583	(89)
		fish	5	0.6	(0.3)	1018	(59)	22.7	(4.5)	1275	(66)
			10	1.4	(0.6)	1159	(77)	26.0	(4.2)	1829	(119)
			20	0.8	(0.4)	1357	(117)	39.1	(5.3)	2582	(160)
		chair	5	0.7	(0.3)	936	(58)	12.3	(2.9)	1228	(82)
			10	0.4	(0.2)	1033	(73)	20.4	(4.2)	1747	(162)
			20	1.2	(0.5)	1167	(91)	35.4	(4.4)	2352	(126)
		ship	5	0.4	(0.3)	819	(43)	5.4	(1.3)	959	(66)
			10	0.6	(0.3)	897	(64)	8.7	(1.6)	1252	(104)
			20	0.6	(0.3)	1004	(79)	12.2	(1.2)	1731	(127)
Exp.2	Upright	chimpanzee	10	0.4	(0.2)	855	(49)	6.5	(1.5)	1211	(60)
		fish	10	1.4	(0.3)	1087	(63)	37.1	(4.2)	1695	(72)
		chair	10	1.4	(0.3)	966	(89)	25.3	(2.0)	1484	(37)
		ship	10	0.4	(0.3)	929	(66)	15.8	(2.1)	1313	(56)
	Inverted	chimpanzee	10	1.1	(0.2)	1024	(98)	14.4	(2.6)	1468	(90)
		fish	10	2.1	(0.6)	1132	(85)	34.1	(2.8)	1789	(85)
		chair	10	2.0	(0.6)	1065	(96)	39.1	(4.0)	1707	(103)
		ship	10	1.1	(0.4)	941	(71)	17.9	(1.5)	1394	(61)
Exp.3	Upright	chimpanzee	10	0.6	(0.2)	902	(63)	10.2	(2.7)	1219	(75)
		chair	10	1.3	(0.4)	1008	(69)	25.3	(2.8)	1530	(76)
		animal	10	0.3	(0.2)	900	(45)	12.3	(2.5)	1316	(62)
		human	10	1.3	(0.4)	1019	(80)	22.9	(5.3)	1561	(110)
Exp.4	Upright	chimpanzee	5	0.0	(0.0)	735	(42)	0.7	(0.7)	811	(21)
			10	0.0	(0.0)	821	(23)	0.7	(0.7)	1029	(58)
			20	0.0	(0.0)	817	(55)	0.7	(0.7)	1312	(66)
		fish	5	0.0	(0.0)	880	(54)	0.7	(0.7)	1178	(64)
			10	0.0	(0.0)	1025	(78)	4.3	(1.6)	1705	(58)
			20	0.7	(0.7)	956	(63)	12.9	(2.8)	2727	(284)
		chair	5	0.0	(0.0)	761	(27)	0.7	(0.7)	966	(71)
			10	0.7	(0.7)	787	(32)	0.7	(0.7)	1302	(66)
			20	0.0	(0.0)	819	(37)	3.6	(0.9)	2235	(162)
		ship	5	0.0	(0.0)	746	(28)	0.0	(0.0)	884	(45)
			10	0.0	(0.0)	769	(34)	0.7	(0.7)	1146	(70)
			20	0.0	(0.0)	776	(20)	0.0	(0.0)	1535	(59)
		human	5	0.7	(0.7)	696	(32)	0.0	(0.0)	771	(34)
			10	0.0	(0.0)	749	(35)	0.7	(0.7)	923	(50)
			20	0.0	(0.0)	822	(28)	0.0	(0.0)	1233	(65)

Note. RT: response times, SE: standard error, SD: same-distractor, DD: different-distractor

Table S2A. Summary of GLMMs for preliminary training: Error rates

	Fixed effects	Levels	Estimate	SE	Z	p	corrected p	95%CI		95%CI (corrected)	
Preliminary training	Target	Chimp vs Fish	0.890	0.286	3.118	1.8E-03	9.1E-03	0.331	1.450	0.155	1.626
Phase 1		Chimp vs Chair	0.413	0.292	1.413	1.6E-01	3.2E-01	-0.160	0.986	-0.242	1.068
Set size=4		Chimp vs Ship	-0.313	0.310	1.007	3.1E-01	3.1E-01	-0.921	0.296	-0.921	0.296
SD trials		Fish vs Chair	-0.477	0.277	1.725	8.5E-02	2.5E-01	-1.020	0.065	-1.140	0.185
		Fish vs Ship	-1.203	0.296	4.064	4.8E-05	2.9E-04	-1.783	-0.623	-1.984	-0.422
		Chair vs Ship	-0.726	0.303	2.392	1.7E-02	6.7E-02	-1.320	-0.131	-1.483	0.032
Phase 2	Target	Chimp vs Fish	1.916	0.292	6.566	5.2E-11	3.1E-10	1.344	2.487	1.146	2.685
Set size varied		Chimp vs Chair	1.293	0.305	4.236	2.3E-05	9.1E-05	0.695	1.891	0.531	2.056
SD trials		Chimp vs Ship	0.907	0.319	2.844	4.5E-03	8.9E-03	0.282	1.531	0.192	1.621
		Fish vs Chair	-0.622	0.200	3.114	1.9E-03	5.5E-03	-1.014	-0.231	-1.101	-0.144
		Fish vs Ship	-1.009	0.220	4.591	4.4E-06	2.2E-05	-1.440	-0.578	-1.575	-0.443
		Chair vs Ship	-0.387	0.238	1.627	1.0E-01	1.0E-01	-0.852	0.079	-0.852	0.079
Phase 3	Target	Chimp vs Fish	2.350	0.548	4.292	1.8E-05	1.1E-04	1.277	3.424	0.905	3.795
Set size=10		Chimp vs Chair	1.728	0.566	3.047	2.3E-03	1.2E-02	0.618	2.838	0.267	3.183
SD trials		Chimp vs Ship	1.469	0.576	2.554	1.1E-02	3.2E-02	0.342	2.597	0.092	2.847
		Fish vs Chair	-0.623	0.312	2.003	4.5E-02	9.0E-02	-1.234	-0.011	-1.325	0.074
		Fish vs Ship	-0.881	0.330	2.672	7.5E-03	3.0E-02	-1.527	-0.235	-1.704	-0.058
		Chair vs Ship	-0.258	0.357	0.715	4.7E-01	4.7E-01	-0.959	0.442	-0.956	0.445
Phase 3	Target	Chimp vs Fish	2.823	0.183	15.454	0	0	2.465	3.181	2.341	3.305
DD trials		Chimp vs Chair	2.786	0.184	15.138	0	0	2.425	3.147	2.312	3.260
		Chimp vs Ship	1.406	0.184	7.638	2.2E-14	4.4E-14	1.045	1.766	0.993	1.818
		Fish vs Chair	-0.037	0.158	0.233	8.2E-01	8.2E-01	-0.346	0.273	-0.346	0.273
		Fish vs Ship	-1.417	0.161	8.815	0	0	-1.732	-1.102	-1.819	-1.016
		Chair vs Ship	-1.381	0.163	8.493	0	0	-1.699	-1.062	-1.770	-0.991

Table S2B. Summary of GLMMs for preliminary training: Response times

	Fixed effects	Levels	Estimate	SE	df	t	p	corrected p	95%CI		95%CI (corrected)	
Preliminary trainin	Target	Chimp vs Fish	0.181	0.035	78.1	5.260	1.2E-06	6.1E-06	0.114	0.249	0.042	0.321
Phase 1		Chimp vs Chair	0.131	0.034	76.9	3.825	2.7E-04	8.0E-04	0.064	0.199	0.038	0.225
Set size=4		Chimp vs Ship	-0.028	0.034	75.5	0.821	4.1E-01	4.1E-01	-0.095	0.039	-0.106	0.050
SD trials		Fish vs Chair	-0.050	0.033	101.2	1.496	1.4E-01	2.8E-01	-0.116	0.015	-0.136	0.035
		Fish vs Ship	-0.210	0.033	99.4	6.303	8.1E-09	4.9E-08	-0.275	-0.144	-0.307	-0.112
		Chair vs Ship	-0.160	0.033	98.1	4.820	5.2E-06	2.1E-05	-0.224	-0.095	-0.252	-0.067
Phase 2	Set size		0.011	0.001	8330.0	14.317	6.0E-46	—	0.009	0.012	—	—
Set size varied	Target	Chimp vs Fish	0.168	0.017	134.1	9.703	3.5E-17	1.7E-16	0.134	0.202	0.119	0.217
SD trials		Chimp vs Chair	0.119	0.017	132.5	6.918	1.8E-10	5.4E-10	0.086	0.153	0.073	0.166
		Chimp vs Ship	-0.008	0.017	132.0	0.479	6.3E-01	6.3E-01	-0.042	0.026	-0.047	0.031
		Fish vs Chair	-0.049	0.017	136.6	2.794	6.0E-03	1.2E-02	-0.083	-0.014	-0.093	-0.005
		Fish vs Ship	-0.176	0.017	136.1	10.149	2.3E-18	1.4E-17	-0.210	-0.142	-0.227	-0.126
		Chair vs Ship	-0.128	0.017	134.4	7.379	1.5E-11	6.0E-11	-0.162	-0.094	-0.176	-0.080
Phase 3	Target	Chimp vs Fish	0.271	0.021	241.8	13.217	2.3E-30	1.4E-29	0.231	0.311	0.179	0.363
Set size=10		Chimp vs Chair	0.214	0.021	240.2	10.319	6.7E-21	2.7E-20	0.173	0.254	0.156	0.270
SD trials		Chimp vs Ship	0.023	0.020	239.0	1.142	2.5E-01	2.5E-01	-0.017	0.063	-0.023	0.069
		Fish vs Chair	-0.057	0.021	244.7	2.812	5.3E-03	1.1E-02	-0.098	-0.017	-0.110	-0.006
		Fish vs Ship	-0.248	0.021	243.5	12.053	1.5E-26	7.7E-26	-0.288	-0.207	-0.306	-0.189
		Chair vs Ship	-0.190	0.021	241.9	9.167	2.2E-17	6.6E-17	-0.231	-0.150	-0.244	-0.134
Phase 3	Target	Chimp vs Fish	0.385	0.027	234.4	14.284	1.1E-33	6.3E-33	0.332	0.437	0.272	0.497
DD trials		Chimp vs Chair	0.371	0.027	230.2	13.588	2.9E-31	1.4E-30	0.317	0.424	0.294	0.448
		Chimp vs Ship	0.048	0.024	157.2	2.004	4.7E-02	9.4E-02	0.001	0.094	-0.012	0.108
		Fish vs Chair	-0.014	0.031	332.4	0.442	6.6E-01	6.6E-01	-0.074	0.047	-0.083	0.056
		Fish vs Ship	-0.337	0.028	258.5	12.111	5.0E-27	2.0E-26	-0.391	-0.282	-0.413	-0.260
		Chair vs Ship	-0.323	0.028	251.6	11.479	8.5E-25	2.6E-24	-0.378	-0.268	-0.398	-0.248

Table S3A. Summary of GLMMs for same-distractor trials in Experiments 1-4: Error rates

	Fixed effects		Estimate	SE	Z	p	corrected p	95%CI		95%CI	
Exp. 1	Target	Chimp vs Fish	1.211	0.527	2.296	2.2E-02	1.3E-01	0.178	2.245	-0.180	2.603
		Chimp vs Chair	1.077	0.533	2.019	4.4E-02	2.2E-01	0.032	2.122	-0.297	2.451
		Chimp vs Ship	0.589	0.567	1.038	3.0E-01	6.0E-01	-0.522	1.700	-0.682	1.860
		Fish vs Chair	-0.134	0.409	0.329	7.4E-01	7.4E-01	-0.936	0.667	-0.936	0.667
		Fish vs Ship	-0.622	0.455	1.367	1.7E-01	6.9E-01	-1.514	0.270	-1.759	0.515
		Chair vs Ship	-0.488	0.462	1.058	2.9E-01	8.7E-01	-1.392	0.417	-1.593	0.617
Exp.2	Trial Type		0.572	0.247	2.315	2.1E-02	—	0.088	1.056	—	—
	Target	Chimp vs Fish	0.877	0.362	2.421	1.6E-02	9.3E-02	0.167	1.587	-0.079	1.833
		Chimp vs Chair	0.815	0.365	2.235	2.5E-02	1.0E-01	0.100	1.530	-0.096	1.726
		Chimp vs Ship	0.028	0.426	0.066	9.5E-01	9.5E-01	-0.807	0.863	-0.807	0.863
		Fish vs Chair	-0.062	0.287	0.215	8.3E-01	1.7E+00	-0.625	0.501	-0.706	0.582
		Fish vs Ship	-0.849	0.362	2.343	1.9E-02	9.6E-02	-1.559	-0.139	-1.782	0.084
		Chair vs Ship	-0.787	0.365	2.159	3.1E-02	9.3E-02	-1.502	-0.073	-1.660	0.086

Note. the null model was selected for the error rate data in Experiment 3.

No statistical analysis was conducted for the data from Experiment 4.

Table S3B. Summary of GLMMs for same-distractor trials in Experiments 1, 2, and 3: Response times

	Fixed effects	Levels	Estimate	SE	df	t	p	correcetd p	95%CI		95%CI (correceted)	
Exp. 1	Set size		0.011	0.001	8364.0	14.803	5.8E-49	—	0.009	0.012	—	—
	Target	Chimp vs Fish	0.239	0.016	272.4	14.792	9.9E-37	5.9E-36	0.207	0.270	0.192	0.285
		Chimp vs Chair	0.152	0.016	271.5	9.432	1.9E-18	7.6E-18	0.120	0.183	0.107	0.196
		Chimp vs Ship	0.016	0.016	271.1	1.018	3.1E-01	3.1E-01	-0.015	0.048	-0.020	0.053
		Fish vs Chair	-0.087	0.016	273.2	5.371	1.7E-07	3.4E-07	-0.119	-0.055	-0.128	-0.046
		Fish vs Ship	-0.222	0.016	272.9	13.692	8.1E-33	4.1E-32	-0.254	-0.190	-0.268	-0.176
		Chair vs Ship	-0.135	0.016	271.9	8.359	3.3E-15	1.0E-14	-0.167	-0.103	-0.178	-0.092
Exp.2	Trial Type		0.065	0.011	4603.0	5.887	4.2E-09	—	0.043	0.087	—	—
	Target	Chimp vs Fish	0.153	0.018	278.0	8.524	1.0E-15	5.0E-15	0.118	0.189	0.103	0.204
		Chimp vs Chair	0.068	0.018	276.2	3.783	1.9E-04	3.8E-04	0.033	0.103	0.023	0.113
		Chimp vs Ship	-0.009	0.018	283.1	0.514	6.1E-01	6.1E-01	-0.044	0.026	-0.049	0.031
		Fish vs Chair	-0.086	0.018	277.3	4.728	3.6E-06	1.4E-05	-0.121	-0.050	-0.136	-0.036
		Fish vs Ship	-0.163	0.018	275.4	8.949	5.5E-17	3.3E-16	-0.198	-0.127	-0.215	-0.110
		Chair vs Ship	-0.077	0.018	273.5	4.255	2.9E-05	8.6E-05	-0.112	-0.041	-0.125	-0.029
Exp.3	Target	Chimp vs. Chair	0.083	0.023	150.6	3.620	4.0E-04	1.6E-03	0.037	0.129	-0.011	0.176
		Chimp vs. Animal	0.008	0.023	140.5	0.364	7.2E-01	7.2E-01	-0.038	0.054	-0.043	0.060
		Chimp vs. Human	0.109	0.023	127.0	4.764	5.1E-06	3.1E-05	0.063	0.155	0.042	0.176
		Chair vs. Animal	-0.074	0.023	150.2	3.261	1.4E-03	4.1E-03	-0.121	-0.029	-0.135	-0.013
		Chair vs. Human	0.026	0.023	135.3	1.156	2.5E-01	5.0E-01	-0.020	0.072	-0.031	0.084
		Animal vs. Human	0.101	0.023	134.0	4.413	2.1E-05	1.0E-04	0.055	0.148	0.036	0.166
Exp.4	Set size		0.005	0.001	2085.0	4.553	5.6E-06	—	0.003	0.007	—	—
	Target	Chimp vs. Fish	0.162	0.021	2085.0	7.676	2.5E-14	2.0E-13	0.120	0.203	0.099	0.224
		Chimp vs. Chair	0.013	0.021	2085.0	0.631	5.3E-01	5.3E-01	-0.028	0.055	-0.034	0.061
		Chimp vs. Ship	-0.014	0.021	2085.0	0.662	5.1E-01	1.0E+00	-0.055	0.027	-0.067	0.039
		Chimp vs. Human	-0.045	0.021	2085.0	2.137	3.3E-02	1.6E-01	-0.086	-0.004	-0.104	0.014
		Fish vs. Chair	-0.149	0.021	2085.0	7.041	2.6E-12	1.8E-11	-0.190	-0.107	-0.210	-0.087
		Fish vs. Ship	-0.176	0.021	2085.0	8.338	1.4E-16	1.2E-15	-0.217	-0.134	-0.239	-0.113
		Fish vs. Human	-0.207	0.021	2085.0	9.807	3.2E-22	3.2E-21	-0.248	-0.165	-0.271	-0.143
		Chair vs. Ship	-0.027	0.021	2085.0	1.292	2.0E-01	5.9E-01	-0.069	0.014	-0.083	0.028
		Chair vs. Human	-0.058	0.021	2085.0	2.766	5.7E-03	3.4E-02	-0.100	-0.017	-0.119	0.002
		Ship vs. Human	-0.031	0.021	2085.0	1.475	1.4E-01	5.6E-01	-0.072	0.010	-0.089	0.027

Table S4. Results of GLMM for perimetric comlexity of each stimulus category

	Estimate	SE	df	t	p	corrected p	95%CI		95%CI (corrected)	
Chimp vs. Fish	-5.11	42.16	1442.0	0.121	9.0E-01	>1	-87.74	77.53	-110.51	100.30
**Chimp vs. Chair**	**-207.10**	**46.97**	**1442.0**	**4.409**	**1.1E-05**	**1.8E-04**	**-299.17**	**-115.04**	**-355.87**	**-58.33**
**Chimp vs. Ship**	**-317.82**	**49.53**	**1442.0**	**6.417**	**1.9E-10**	**3.7E-09**	**-414.89**	**-220.75**	**-477.88**	**-157.75**
Chimp vs. Animal	-52.36	63.69	1442.0	0.822	4.1E-01	>1	-177.18	72.46	-226.69	121.96
Chimp vs. Human	-52.40	57.96	1442.0	0.904	3.7E-01	>1	-166.01	61.21	-218.68	113.87
**Chimp vs. DST**	**-167.79**	**32.20**	**1442.0**	**5.211**	**2.1E-07**	**3.9E-06**	**-230.89**	**-104.68**	**-270.87**	**-64.71**
**Fish vs. Chair**	**-202.00**	**45.13**	**1442.0**	**4.475**	**8.2E-06**	**1.4E-04**	**-290.46**	**-113.53**	**-345.74**	**-58.25**
**Fish vs. Ship**	**-312.71**	**47.79**	**1442.0**	**6.544**	**8.2E-11**	**1.7E-09**	**-406.37**	**-219.05**	**-467.82**	**-157.60**
Fish vs. Animal	-47.26	62.34	1442.0	0.758	4.5E-01	>1	-169.45	74.93	-211.90	117.38
Fish vs. Human	-47.30	56.49	1442.0	0.837	4.0E-01	>1	-158.01	63.41	-206.03	111.43
**Fish vs. DST**	**-162.68**	**29.45**	**1442.0**	**5.524**	**3.9E-08**	**7.4E-07**	**-220.40**	**-104.95**	**-257.43**	**-67.93**
Chair vs. Ship	-110.72	52.08	1442.0	2.126	3.4E-02	3.0E-01	-212.80	-8.64	-266.70	45.26
Chair vs. Animal	154.74	65.69	1442.0	2.356	1.9E-02	2.0E-01	25.98	283.49	-46.01	355.49
Chair vs. Human	154.70	60.16	1442.0	2.571	1.0E-02	1.2E-01	36.78	272.61	-30.72	340.12
Chair vs. DST	39.32	36.00	1442.0	1.092	2.8E-01	>1	-31.25	109.88	-65.70	144.34
**Ship vs. Animal**	**265.46**	**67.54**	**1442.0**	**3.930**	**8.9E-05**	**1.2E-03**	**133.07**	**397.84**	**54.20**	**476.72**
**Ship vs. Human**	**265.42**	**62.18**	**1442.0**	**4.269**	**2.1E-05**	**3.1E-04**	**143.55**	**387.28**	**69.66**	**461.18**
**Ship vs. DST**	**150.03**	**39.28**	**1442.0**	**3.820**	**1.4E-04**	**1.8E-03**	**73.05**	**227.02**	**28.03**	**272.03**
Animal vs. Human	-0.04	73.95	1442.0	0.001	1.0E+00	1.0E+00	-144.98	144.90	-165.92	165.84
Animal vs. DST	-115.42	56.09	1442.0	2.058	4.0E-02	3.2E-01	-225.35	-5.49	-281.37	50.53
Human vs. DST	-115.38	49.49	1442.0	2.331	2.0E-02	2.0E-01	-212.39	-18.37	-265.21	34.45

Note. Rows are shown in bold if the corrected CIs do not include zero.
